# Intracranial Venous Pressures Manometry for Patients With Idiopathic Intracranial Hypertension: Under Awake Setting or General Anesthesia

**DOI:** 10.3389/fneur.2019.00751

**Published:** 2019-07-12

**Authors:** Xin-bin Guo, Sen wei, Sheng Guan

**Affiliations:** Department of Interventional Radiology, The First Affiliated Hospital of Zhengzhou University, Zhengzhou, China

**Keywords:** venous sinus, idiopathic intracranial hypertension, pressure gradient, stenosis, stent

## Abstract

**Background and Purpose:** Venous sinus stenting (VSS) is a well-acknowledged treatment strategy for patients with a high venous sinus pressure gradient across the site of outflow obstruction. It is not clear whether intracranial venous pressure manometry should be performed awake or under general anesthesia (GA). The aim of this study is to compare the accuracy of venous manometry performed under GA or awake setting, and to evaluate stenting candidates to be determined under awake setting or under GA.

**Methods:** The manometry results of 32 patients with idiopathic intracranial hypertension (IIH) were recorded under awake setting and general anesthesia before stenting. Mean venous pressures (MVPs) and trans-stenosis pressure gradients were obtained and compared between awake setting and general anesthesia status.

**Results:** MVPs and trans-stenosis pressure gradients of 32 patients under GA and awake pressure setting were recorded. MVPs in the superior sagittal sinus, torcula, and transverse sinus were lower in the GA group, without statistical significant difference (*P* > 0.05). MVPs were significantly higher in the sigmoid sinus and jugular bulb under GA group (*p* < 0.05). Mean trans-stenosis pressure gradient was significantly lower in the group under GA (*p* < 0.05).

**Conclusions:** Intracranial venous pressure seems to be affected by different levels of consciousness. Our study reveals that intracranial venous pressure is lower under general anesthesia than in the awake setting, which may have a potential impact on patient selection for venous sinus stenting.

## Introduction

Idiopathic intracranial hypertension (IIH) is a syndrome characterized by headaches, visual obscurations, and elevated intracranial pressure (ICP). Although the underlying pathophysiology of IIH is still unclear, venous sinus stenosis with increased trans-stenosis pressure gradient caused by cerebral venous congestion has been frequently identified as a contributing factor (1, 2). Therefore, venous sinus stenting (VSS) has been recognized as a popular treatment option for patients with IIH. Specific patients with symptomatic VSS without IIH may also benefit from stenting (3–6). The available literatures suggest that patients with venous sinus outflow obstruction behave as those with central cerebral venous hypertension, which is associated with sinus stenosis or occlusive arachnoid granulations. As a result, successful patient selection for further management mainly depends on accurate venous pressure measurements. VSS for patients with concomitant IIH and intracranial venous stenosis has been shown in retrospective series to reduce ICP, improve visual outcomes, and ameliorate headaches and tinnitus. Trans-stenosis gradient pressure is used as one of the valuable indicators to assist in making treatment decisions. A pressure gradient of 8–10 mm Hg across intracranial venous sinus stenosis has been identified as a threshold for intervention treatment in most series (3–7).

To treat vision and headache deterioration with interventional therapy, routine practice is to evaluate venous pressures before stenting, while VSS is typically interventionally treated under GA. Until now, the effects of awake setting or GA on venous pressure measurements appear to be debatable, and the studies have not been widely reported (8, 9). Therefore, whether intracranial venous pressures should be measured under awake setting or under GA for patients with IIH before stenting is still not clear. In this study, we measured venous pressure under awake setting and GA before VSS stenting, with the aim to quantify the anesthesia impact on MVPs and the trans-stenosis pressure gradient. In our cohort, we compared the venous sinus pressures measured under awake setting and GA before stenting, and speculate how stenting candidates should be determined under awake setting.

## Subjects and Methods

A retrospective, single-center review of patients with IIH was conducted from January 2010 to January 2019 after the approval of the institutional review board. Informed consent was obtained from all individual participants included in the study. Venous manometry results from 32 intracranial venous sinus stenosis patients who had undergone diagnostic angiography were obtained. Patients with a trans-stenosis gradient of ≥ 8 mmHg were included, and subsequently underwent venous sinus stenting under GA. A database of patients who underwent VSS during this time period was recorded, while the subgroup of patients under awake diagnostic cerebral angiogram with manometry before the stenting procedure were identified. In this subgroup, venous manometry was performed during awake angiogram. After that, stent deployment was conducted under GA.

Medical charts, imaging findings, and anesthetic reports were reviewed to evaluate patients' demographic information, intra-procedural findings, and details of administered anesthetic medication. Imaging reports were reviewed to assess MVPs recorded at various locations throughout the venous sinus system for comparison between cases performed under awake setting vs. GA.

## Venography and venous manometry Under Awake Setting

All pressure measurements were immediately recorded by independent imaging technicians. Angiography, venography, and venous sinus manometry were performed on patients who were awake with no conscious sedation. In all patients, the femoral artery and vein were both accessed by 6 F sheaths(Terumo Corporation, Japan). A 5F diagnostic catheter (Terumo Corporation, Japan) was used to perform a cerebral arteriogram to evaluate venous outflow pathways. Next, a 6F diagnostic catheter (Terumo Corporation, Japan) was advanced into the dominant internal jugular vein (IJ). A Rebar-27 microcatheter (Medtronic, Minneapolis, Minnesota, USA) was navigated over a 0.014 inch microwire (Traxcess 14, Micro-Vention, Inc. USA) into the superior sagittal sinus (SSS). A diagnostic cerebral venogram was then performed followed by serial venous manometry measurements in the SSS, torcula, transverse sinus (TS), sigmoid sinus (SS), and ipsilateral JV. At each location, the pressure was stabilized before recordings were made. When possible, bilateral transverse–sigmoid pathways were accessed, and manometry recording were performed on both sides.

## Manometry Before Venous Sinus Stenting Under Anesthesia

Venous sinus stenting was performed under GA. Aspirin and clopidogrel were administered before the procedure in each patient. After the induction of GA, the right femoral vein was accessed by an 8 F sheath (Terumo Corporation, Japan), and IV heparin was administered. Pre-stenting manometry was variably performed. A Rebar-27 microcatheter was used to measure ipsilateral venous pressures. After manometry, stenting was performed under standardized procedure.

## Assessment of Outcomes and Statistical Analysis

To test pressure differences between awake and GA by location and in total, descriptive statistics comparing MVPs and pressure gradients at various locations were firstly generated. Data are presented as mean and SD for continuous variables. Pressure estimations are presented by location for awake, GA, and the difference between them. For all analyses, unpaired student‘s *t-*testing was used with *P* < 0.05, interpreted for statistical significance.

## Results

### Patient Characteristics

From January 2010 to January 2019, the result of 32 IIH patients who had undergone diagnostic angiography and venous manometry for intracranial venous sinus stenosis investigation were obtained. Venous sinus pressure measurements were obtained from the above patients under awake setting before stenting was performed under GA. The mean age of these patients is 37.2 years old (range 21–53 years), and 91.4% of the study group is female. Median body mass index (BMI) is 28.6 kg/m^2^ (range 24.2–46.3 kg/m^2^). Median opening pressure on lumbar puncture was 38.6 cm H2O (range 29–57 cm H_2_O). Demographic and procedural details for the 32 patients are shown in Table 1. Pressure measurements of four locations were recorded in each patient.

**Table 1:** Patient characteristic awake setting before stenting.

**Patient characteristics**	***N* = 32**
Female	28
Mean age (y)	37.2
BMI (kg/m^2^)	28.6
CSF pressure (cmH_2_O)	38.6
**Stented segment**	
Right transverse sinus	22 (68.8%)
Left transverse sinus	9 (28.1%)
Superior sagittal sinus	1 (3.1)

### Location Comparison Under Awake vs. GA

**Figure 1:**
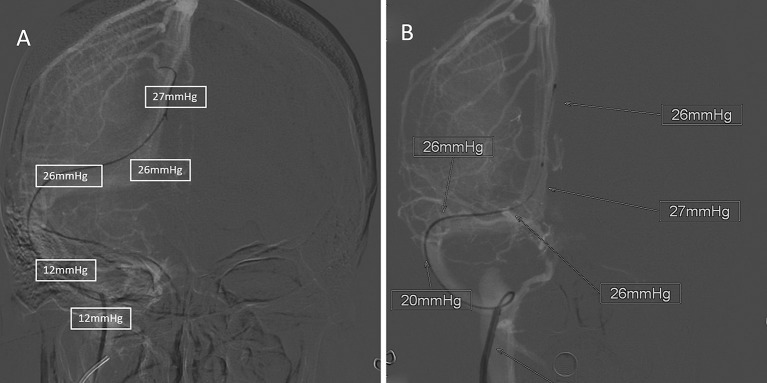
A typical 32-year-old female patient with IIH. All pressure outcomes were immediately marked by the imaging technician. **(A)** was the recording of pressure outcome under awake setting, and **(B)** was the recording of pressure outcome under GA.

All pressure measurements were immediately recorded by independent imaging technicians (Figure 1). For all patients, MVPs at various locations in the intracranial venous circulation under awake setting vs. GA are shown in Table 2. A significant pressure difference between awake and GA was detected in the SS (*p* < 0.05). MVPs were significantly different between awake setting and GA in SS (*p* < 0.05), and MVPs were significantly lower under awake setting than GA in the SS.

**Table 2:** Comparison of awake with general anesthesia venous pressures by location.

	**Awake**	**Anesthesia**	
**Location**	**Mean (SD)**	**Mean (SD)**	***p*-value**
Superior sagittal sinus	36.19 (8.63)	35.15 (9.46)	0.47
Torcula	35.54 (9.72)	34.76 (10.35)	0.59
Transverse sinus	36.89 (8.45)	35.04 (9.04)	0.19
Sigmoid sinus	12.90 (5.39)	15.26 (4.30)	0.038
Gradient	22.71 (7.76)	19.20 (8.53)	0.020

There was no statistically significant difference between awake and general in the SSS, torcula and TS (*p* > 0.05) (Table 2). MVPs were higher under awake setting than GA in the SSS, torcula, and TS with no statistical significance, and MVPs were significantly lower (*p* < 0.05) under awake setting compared with GA in the SS. Table 3 shows the absolute magnitude of pressure differences for patients awake and under GA, according to location. For all patients, pressure of 18/32 patients (56.3%) were higher when performed under GA compared with awake setting in SSS with no statistical significance (*p* = 0.47), and 81.3% of SS pressure were significantly higher (*p* = 0.038) under GA compared with awake setting (Table 3).

**Table 3:** The absolute difference between venous pressure measurements under awake and when general anesthesia by location (*N* = 32).

	**Decreased by**	**Decreased by**	**Increased by**	**Increased by**
	**10+ mmHg**	**0–10 mmHg**	**0–10 mmHg**	**10+ mmHg**
**Location**	***N* (%)**	***N* (%)**	***N* (%)**	***N* (%)**
Superior sagittal sinus	1 (3.1)	13 (40.6)	16 (50)	2 (6.3)
Torcula	1 (4.2)	12 (37.5)	17 (53.1)	3 (6.3)
Transverse sinus	2 (6.3)	11 (39.3)	16 (50)	3 (9.4)
Sigmoid sinus	1 (3.1)	5 (15.6)	25 (78.2)	1 (3.1)
Gradient	4 (12.5)	22 (68.8)	5 (15.6)	1 (3.1)

### Pressure Gradients Under Awake Setting vs. GA

**Figure 2:**
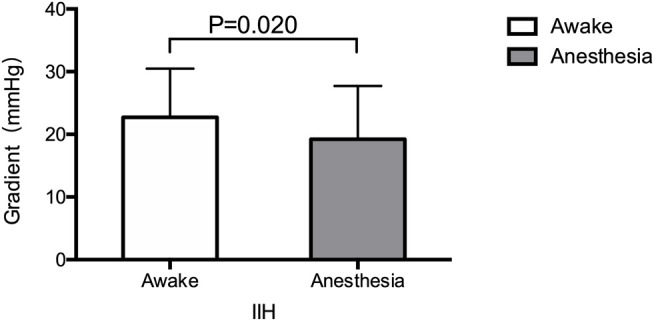
The trans-stenosis pressure gradients under awake setting and general anesthesia (GA) before stenting. Pressure gradient are significantly lower under GA than under awake setting (*p* < 0.05).

All patients underwent angiography in awake setting before VSS under anesthesia. Trans-stenosis pressure gradient was significantly lower under GA (*p* < 0.05) (Figure 2). The mean pressure gradient was 22.71 mmHg under awake setting, compared with 19.20 mmHg under GA. Pressure gradients of ≥8 mmHg are considered diagnostic for venous stenosis, while in our cohort, all 32 pressure gradients were ≥8 mmHg under awake setting. Of these patients, 22/32 (68.8%) pressure gradients decreased by 0–10 mmHg under GA, and 4/32 (12.5%) had pressure gradients <8 mmHg under GA (Table 3). The pressure gradient decrease reached a statistically significant difference in patients under GA. Since almost all pressure gradients decreased under GA compared with the awake setting, we speculate that gradients under GA might mislead the therapeutic decision of stenting.

## Discussion

For patients with IIH who are refractory to medical therapy, stenting treatment indications for VSS generally include: radiographic evidence of intracranial venous sinus stenosis; and a trans-stenosis pressure gradient of at least 8–10 mm Hg (3–11). MVPs and pressure gradients are integral parameters for diagnostic components for VSS, which provide baseline information for endovascular therapeutic decisions potentially benefiting the patients. This study identified venous manometry differences between awake and GA before VSS. Specifically, MVPs are significantly increased under GA in the SS (*p* < 0.05), while without significant difference in the SSS, torcula, and TS (*p* > 0.05). Trans-stenosis pressure gradient is significantly decreased under GA, which might indicate that the gradients under GA may not be a reliable result for the therapeutic decision of stenting.

Fargen et al. examined the effect of anesthesia on venous pressure readings among patients undergoing VSS (8), they found a high variation in venous pressure measurements between awake setting and GA, but overall measurements were higher under GA. Raper etal. examined the effects of Intracranial venous pressures under conscious sedation and GA (9). In this study, the MVPs in the superior sagittal sinus, torcula, and transverse sinus were lower under GA, but were significantly higher in the sigmoid sinus under GA (*p* < 0.001). Furthermore, they found that MVPs consistently decreased under GA compared with awake in the TS and SS. In our study, the results show that MVPs were lower under GA than under awaking setting in the SSS and TS with no significant difference(*p* > 0.05), and there was a high variation in venous pressure measurements between awake setting and GA. The differences among different cohorts are not entirely clear, and needs further investigation.

Most neurointerventionalists consider the pressure gradient across the region of obstruction as the principal factor determining candidacy for VSS, with a threshold of ≥8 mm Hg. Our study indicates that the gradients performed under GA are likely to vary more from those obtained while patients are awake. In most situations, anesthesia appears to exaggerate the SS pressure. Almost all gradients were lower when obtained under GA compared with the awake setting (12.5% of the patients had pressure gradients <8 mm Hg under GA). These data therefore suggest that pressure measurements obtained under anesthesia maybe unreliable and might falsely rule out patients for stenting procedure.

Our patients generally underwent awake and anesthesia procedures using similar agents to those reported by Fargen et al. Although venous pressures obtained in the SS under awake were generally higher, while 6/32 (18.7%) of patients had a reduction in pressures under anesthesia compared with awake. The reasons for such variability are unclear. Despite these differences between cohorts, number of other factors could potentially affect venous pressure, causing differences between anesthetic level and among individual patients. For example, obstructive sleep apnea and other respiratory disorders can lead to significant variation in ICP, owing to hypoxic and hypercapnic cerebral vasodilatation (12–14). The end-tidal CO_2_ changes had a dramatic effect on the mean pressure on venous pressure measurements (15), but other factors causing variation between anesthetic levels are unclear.

Limitations of this study are partially related to the retrospective design. Although the approach to awake and GA was consistent, GA was performed by different anesthesiologists; therefore, the aesthetical agents, dosage and surgical skills varied among proceduralists. Data regarding end-tidal CO_2_ throughout VSS were not available for review, which might have had minor effects in MVP findings. Blood pressure recordings were variable and could not be accurately obtained at the time of manometry.

## Conclusions

In summary, this retrospective study evaluated venous sinus manometry measurements obtained under both awake and GA situations. The results show that MVP was significantly affected under GA and majority of the gradients were reduced under GA. Sine pressure gradient critically affects the therapeutic decision of stent indications; our findings argue that candidates for stenting should be determined with venous manometry measured in awake setting instead of GA, in order to avoid the unpredictable and highly variable effect of GA on pressure measurements.

## Data Availability

All datasets generated for this study are included in the manuscript and/or the supplementary files.

## Ethics Statement

All procedures performed in studies involving human participants were in accordance with the ethical standards of Zhengzhou University. Informed consent was obtained from all individual participants included in the study.

## Author Contributions

XG designed the research. XG and SG performed the research. XG wrote the manuscript. All authors discussed and contributed to the analysis of the experimental data.

### Conflict of Interest Statement

The authors declare that the research was conducted in the absence of any commercial or financial relationships that could be construed as a potential conflict of interest.
